# Population structure and demographic history of a tropical lowland rainforest tree species *Shorea parvifolia* (Dipterocarpaceae) from Southeastern Asia

**DOI:** 10.1002/ece3.284

**Published:** 2012-07

**Authors:** Hiroko Iwanaga, Kosuke M Teshima, Ismael A Khatab, Nobuyuki Inomata, Reiner Finkeldey, Iskandar Z Siregar, Ulfah J Siregar, Alfred E Szmidt

**Affiliations:** 1 Department of Biology, Graduate School of Sciences, Kyushu UniversityFukuoka, Japan; 2 Laboratory of Plant Genetics, Graduate School of Agriculture, Kyoto UniversityKyoto, Japan; 3 Department of Genetics, Faculty of Agriculture, Kafr El-Sheikh UniversityKafr El-Sheikh, Egypt; 4 Department of Environmental Science, International College of Arts and Sciences, Fukuoka Women's UniversityJapan; 5 Institute Forest Genetics and Forest Tree Breeding, Georg-August-University of GöttingenGöttingen, Germany; 6 Department of Silviculture, Faculty of Forestry, Bogor Agricultural UniversityBogor (IPB), Indonesia

**Keywords:** Glacial periods, nuclear genes, phylogeography, *Shorea*, Southeastern Asia

## Abstract

Distribution of tropical rainforests in Southeastern Asia has changed over geo-logical time scale, due to movement of tectonic plates and/or global climatic changes. *Shorea parvifolia* is one of the most common tropical lowland rainforest tree species in Southeastern Asia. To infer population structure and demographic history of *S. parvifolia*, as indicators of temporal changes in the distribution and extent of tropical rainforest in this region, we studied levels and patterns of nucleotide polymorphism in the following five nuclear gene regions: *GapC*, *GBSSI*, *PgiC*, *SBE2*, and *SODH*. Seven populations from peninsular Malaysia, Sumatra, and eastern Borneo were included in the analyses. STRUCTURE analysis revealed that the investigated populations are divided into two groups: Sumatra-Malay and Borneo. Furthermore, each group contained one admixed population. Under isolation with migration model, divergence of the two groups was estimated to occur between late Pliocene (2.6 MYA) and middle Pleistocene (0.7 MYA). The log-likelihood ratio tests of several demographic models strongly supported model with population expansion and low level of migration after divergence of the Sumatra-Malay and Borneo groups. The inferred demographic history of *S. parvifolia* suggested the presence of a scarcely forested land bridge on the Sunda Shelf during glacial periods in the Pleistocene and predominance of tropical lowland rainforest at least in Sumatra and eastern Borneo.

## Introduction

Distribution of tropical rainforests in Southeastern Asia has changed over geological time scale, due to movement of tectonic plates and/or global climatic changes (Morley 2000). These changes were likely to affect population structure and genetic variation of rainforest organisms, and as a consequence, their evolution and speciation. Southeastern Asia differs from the other two major rainforest centers in Africa and South America because the distribution of its rainforests has been affected not only by climate, but also by successive submerging and reexposure of the continental shelf resulting from the sea level changes.

Recent studies revealed considerable fluctuations in the distribution and extent of tropical rainforests in this area coinciding with glacial cycles in the Pleistocene (Cannon et al. 2009; Wurster et al. 2010). At the Last Glacial Maximum (LGM; approximately 0.02 MYA), peninsular Malaysia, Sumatra, Java, and Borneo islands were connected by the exposed Sunda Shelf (Voris 2000). Many studies suggested that at this period, rainforest refugia were present in the northern and eastern Borneo, northern and western Sumatra, and Mentawai Islands (Thomas 2000; Gathorne-Hardy et al. 2002; Cannon et al. 2009; Wurster et al. 2010). However, the extent of rainforests in the Sunda region in that period is controversial. Some studies suggested a savanna corridor across the Sunda Shelf separated rainforests of Sumatra-Malay area and Borneo (Kaars 1991; Kaars and Dam 1995; Bird et al. 2005). On the other hand, other studies indicated predominance of tropical rainforest on the Sunda Shelf (Sun et al. 2000; Wang et al. 2009; Cannon et al. 2009).

Phylogeographic analysis, which deals with the geographic distribution of genetic variation, is a powerful tool to infer past demographic events that include population expansion or bottlenecks as well as population structure and migration. Few studies of tropical tree species have been performed on a regional scale in Southeastern Asia. Two montane rainforest tree genera (*Lithocarpus*: Cannon and Manos 2003; *Trigonobalanus*: Kamiya et al. 2002) and a genus of pioneer trees of lowland rainforest (*Macaranga*: Bänfer et al. 2006) were studied using chloroplast (cp) DNA sequences. Canopy tree species of lowland rainforests occupy different ecological niche than montane and pioneer species and are therefore expected to have different demographic history and population structure. So far, only few such species were included in population genetic studies (*Shorea leprosula*: Lee et al. 2000; *S. leprosula* and *S. parvifolia*: Cao et al. 2006). Divergence of Sumatra-Malay and Borneo populations was often demonstrated in these studies. Furthermore, shared polymorphisms between populations were observed in *S. parvifolia* and some *Macaranga* species suggesting gene flow and/or maintenance of the ancestral polymorphism. These two events have completely different historical causes. The former attributes to migration or admixture between formerly separated populations, while the latter attributes to recent divergence of populations.

Evaluation of the levels and patterns of nucleotide polymorphism in multiple nuclear loci is useful in inferences of population structure and estimations of demographic parameters, as well as in detecting natural selection (e.g., Haddrill et al. 2005; Wright and Gaut 2005). Studies on nucleotide polymorphism in nuclear gene regions in tropical tree species from Southeastern Asia are very scarce and limited to a local scale. Nucleotide variation of the nuclear loci *GapC* and *PgiC* of four *Shorea* species (*S. acuminata*, *S. curtisii*, *S. leprosula*, and *S. parvifolia*) in peninsular Malaysia was investigated by Ishiyama et al. (2003) and Ishiyama et al. (2008), respectively. Negative Tajima's *D* values in *S. curtisii*, *S. leprosula*, and *S. parvifolia* and relatively low levels of population differentiations in *S. acuminata* and *S. curtisii* suggested recent expansion of the investigated species in peninsular Malaysia. This result posed new questions about the regional scale of population structure and demographic histories of these species.

**Figure 1:**
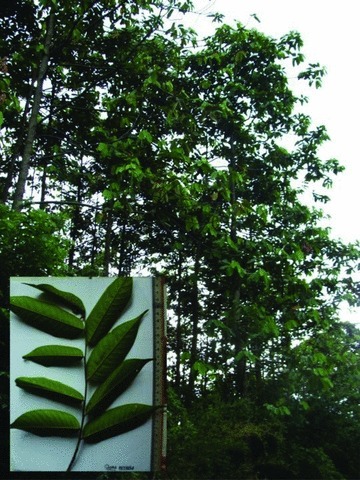
Plantation of *S. parvifolia* and other *Shorea* species in Haurbentes Experimental Garden, Bogor, Indonesia. Inset: twig and leaves of S. *parvifolia*.

**Figure 2:**
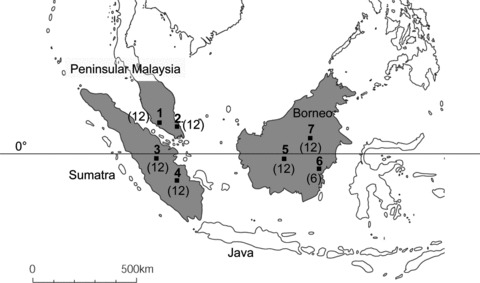
Locations of the sampled populations. Distribution of *Shorea parvifolia* is marked in gray. The numbers indicate populations sampled in this study: 1. Seremban, 2. Mersing, 3. Nanjak Makmur, 4. Asialog, 5. Sari Bumi Kusuma, 6. ITCI Karya Utama, 7. Sumalindo. Numbers in brackets indicate sample size.

In this study, we analyzed levels and patterns of nucleotide polymorphism in five nuclear gene regions from seven populations of *Shorea parvifolia* Dyer collected from peninsular Malaysia, Sumatra, and eastern Borneo. *Shorea parvifolia* is one of the most abundant canopy tree species in lowland evergreen rainforest in Southeastern Asia and distributed throughout peninsular Malaysia, Sumatra and Borneo (Symington 1943; Figs. 1 and 2). Therefore, demographic history of this species could be a good indicator of temporal changes in the distribution of tropical lowland rainforests. Our objectives were (1) to infer population structure and demographic history of this species as indicators of temporal changes in the distribution and extent of tropical rainforest in Southeastern Asia and (2) to determine whether natural selection is acting on the investigated loci.

## Materials and Methods

### Plant materials

Information about the locations and sample sizes of the investigated populations is summarized in Figure 2 and Table S1. Leaf samples of adult trees and saplings were collected from seven populations of *S. parvifolia* including two populations from peninsular Malaysia (Seremban and Mersing), two populations from Sumatra (Nanjak Makmur and Asialog), and three populations from eastern Borneo, Kalimantan (Sari Bumi Kusuma, ITCI Karya Utama, and Sumalindo). Twelve individuals were analyzed for each population, except for the population ITCI Karya Utama, where only six individuals could be sampled. In total, 78 individuals of *S. parvifolia* were analyzed in this study. Most individuals from the population Seremban were the same as those studied by Ishiyama et al. (2003) and Ishiyama et al. (2008). Individuals from populations Nanjak Makmur, Asialog, and Sari Bumi Kusuma were the same as those studied by Cao et al. (2006). Species identification in the field was done based on leaf morphological characters (e.g., length, petiole length, width, distance from petiole to the widest part of the leaf, number of venations, number of lobes, domatia length, and leaf shape).

In our previous studies, one putative hybrid between *S. parvifolia* and *S. acuminata* and one putative hybrid between *S. parvifolia* and *S. leprosula* were found among *S. parvifolia* individuals from peninsular Malaysia based on genotypes of the *GapC* and the *PgiC* gene regions (Ishiyama et al. 2003; Ishiyama et al. 2008). To detect putative interspecific hybrids in populations analyzed in the present study, one individual of each *S. acuminata*, *S. leprosula*, and *S. curtisii*, which belong to the same timber group (Red Meranti) as *S. parvifolia* (Symington 1943) were included. Close relationships of these species have been reported by Kamiya et al. (2005). In addition, one individual of *S. maxwelliana*, which is not closely related with the aforementioned species, was included as outgroup in some neutrality tests. Individuals from these four species were the same as those used by Ishiyama et al. (2003) or Ishiyama et al. (2008).

### Loci studied

Several enzymes that commonly exist in plants and whose functions are well known were chosen as candidate loci for this study. Trial PCR primers were designed based on the known sequences of *Shorea* species obtained in other studies (*GapC*: Ishiyama et al. 2003; *GBSSI*: Kamiya, pers. commun.; *PgiC*: Ishiyama et al. 2008) and expressed sequence tag (EST) data from *S. leprosula* (Tsumura, pers. commun.). Sequences of the PCR products amplified using these primers were determined. Gene regions that contained single-nucleotide repeats (>10) or many indels were excluded. Specific primers for the remaining gene regions were designed based on the sequences of *S. parvifolia* obtained during trial PCR amplifications. Finally, the following five nuclear genes were used: *GapC* (glyceraldehyde-3-phosphate dehydrogenase, EC 1.2.1.12), *GBSSI* (granule-bound starch synthase I, EC 2.4.1.242), *PgiC* (cytosolic glucose-6-phosphate isomerase, EC.5.3.1.9), *SBE2* (starch branching enzyme class II, EC 2.4.1.18), and *SODH* (sorbitol dehydrogenase, EC 1.1.1.14). Names of all loci were assigned according to the corresponding homologues of *Arabidopsis thaliana*.

### DNA isolation, amplification, and sequencing

Genomic DNA was isolated from ∼300 mg of leaves using a modified cetyl trimethyl ammonium bromide method (Murray and Thompson 1980) or DNeasy 96 Plant Kit (QIAGEN). Partial regions of the five nuclear genes were amplified for each individual by PCR. When the efficiency of PCR amplification was poor, nested PCR was performed. Sequences of the primers for PCR, nested PCR, and sequencing are listed in Table S2. PCR amplification conditions were as follows: 35 cycles of denaturation at 94°C for 30 sec, annealing at 55°C for 30 sec and extension at 72°C for 150 sec. For nested PCR, the number of cycles ranged from 15 through 35 according to amplification efficiency. Amplification products were purified using Wizard® SV Gel, and PCR Clean-Up System kit (Promega). Purified products were directly sequenced for both strands using ABI Prism 3100 automatic sequencer (Applied Biosystems). We obtained sequences of both haplotypes for each locus and each individual. When sequences obtained by direct sequencing had no or only one heterozygous site, sequences of both haplotypes of an individual could be directly inferred. On the other hand, when two or more heterozygous sites or indels were detected by direct sequencing, purified amplification products were cloned into the pGEM T-easy vector (Promega). Individual clones were sequenced using universal primers T7 and SP6 for the promoter sites of the vector. To eliminate PCR errors, we carried out the following analyses: we determined sequences of individual clones, until three clones with the same phase at heterozygous sites were obtained. The consensus sequence of these three clones was regarded as a sequence of the first haplotype. This procedure was then repeated using additional clones to obtain the sequence of the second haplotype. Sequences of the obtained haplotypes were compared to the corresponding direct sequence to check consistency. Sequences obtained in this study have been deposited in GenBank with the following accession numbers: AB724403 through AB725191. We also used sequences of the *PgiC* gene region from population Seremban of *S. parvifolia* obtained by Ishiyama et al. (2008), and *PgiC* sequence for one individual of *S. maxwelliana* obtained by Kamiya et al. (2005).

### Data analyses

DNA sequences were verified and assembled into a contiguous sequence for each locus of each individual using the ATGC program ver. 4 (GENETYX CORPORATION). Multiple sequence alignment for individual loci was performed using the Clustal W program ver. 1.4 (Thompson et al. 1994) and corrected manually. Alignment gaps were excluded in all analyses. To assess levels of nucleotide polymorphism, nucleotide diversity (π; Nei 1987) and haplotype diversity (*Hd*; Nei 1987) for each of the five investigated loci were estimated. Population recombination parameter ρ (ρ = 4*N*_e_*c*, where *N_e_* is the effective population size and *c* is the recombination rate per generation per site) was estimated for each locus using the composite-likelihood method (Hudson 2001) implemented in the software package LDhat (http://www.stats.ox.ac.uk/~mcvean/LDhat/index.html). To test for deviation from selective neutrality and other assumptions (random mating, constant population size, and no migration), Tajima's *D* (Tajima 1989) test was performed for individual loci. For this test, the 95% confidence interval of Tajima's *D* statistics of individual loci was obtained using 10,000 replicates of coalescent simulations under standard neutral model (Hudson 1990) with no recombination. The observed number of polymorphic sites was given in coalescent simulations to define the number of mutations. Heterogeneity of the ratio of divergence to polymorphism between synonymous and nonsynonymous sites was tested using the MK test (McDonald and Kreitman 1991) and among the loci by the multilocus HKA test (Hudson et al. 1987). The ratio should be the same, if the tested sites or loci evolve neutrally. *Shorea maxwelliana* was used as an outgroup species in the MK and HKA tests. The multilocus Tajima's *D* test and HKA test were performed using the HKA program obtained from Jody Hey's website (http://lifesci.rutgers.edu/~heylab/). In the multilocus Tajima's *D* test, *P*-values for average Tajima's *D* statistic over five loci were obtained. All calculations and coalescent simulations (except for estimation of ρ and multi-locus Tajima's *D* and HKA tests) were performed using the DnaSP program ver. 4.10.9 (Rozas et al. 2003).

To investigate the degree of population differentiation, fixation indices (*F*_ST_; Hudson et al. 1992) between populations were estimated for each gene region. To visualize relationships of the investigated populations, we constructed neighbor-joining (NJ) tree (Saitou and Nei 1987) based on the net number of nucleotide differences (*Da*; Nei 1987). The tree was constructed using MEGA5 program (Tamura et al. 2011). Furthermore, we used model-based clustering algorithm (Pritchard et al. 2000) implemented in the STRUCTURE program ver. 2.2 (http://pritch.bsd.uchicago.edu/structure.html) to detect population structure and assign individuals to populations. Related haplotypes were grouped and treated as single alleles. Haplotype grouping was performed using the TCS program ver. 1.18 (Clement et al. 2000). All model parameter values in the STRUCTURE analysis were defaults of the program. We conducted five independent simulations with 50,000 iterations for the burn-in phase and 200,000 iterations for the data collection phase. The number of distinct clusters (*K*) was selected based on the Δ*K* statistic of Evanno et al. (2005).

Our STRUCTURE analysis revealed two genetically distinct groups of populations: the Sumatra-Malay group and the Borneo group. To infer history of the splitting event of these population groups, we used the IMa program (Hey and Nielsen 2007). We estimated the following six parameters: 4*N*_e_*u* of Sumatra-Malay group (θ_sm_), Borneo group (θ_b_), and their ancestral population (θ_A_), migration rate from Borneo group to Sumatra-Malay group (*m*_sm_), migration rate from Sumatra-Malay group to Borneo group (*m*_b_), and divergence time (*t*). The program implements Markov chain Monte Carlo simulations for generating genealogy fitting the ‘‘isolation with migration’’ (IM) model (Hey and Nielsen 2004) to data from multiple loci. The infinite-site model (Kimura 1969) was used as mutation model for all loci in the simulations. Since the IM model assumes no recombination, we used the longest part of sequence alignment that showed no evidence of recombination in the four-gamete test (Hudson and Kaplan 1985). First, the prior interval of parameters was obtained empirically by preliminary running IMa program with large parameter intervals. Subsequently, 100,000,000 steps of simulation saving a genealogy for every 1000 steps after a burn-in period (100,000 steps) with the obtained prior maxima of the parameters were performed. Peaks of the resulting marginal posterior probability distributions were defined as estimates of the parameters. Since selective neutrality for the *GBSSI* gene was rejected by the MK test, another simulation was run with four loci excluding this locus to check how estimates are affected by this locus.

The six parameters estimated by the IMa program were converted to the actual demographic parameters (i.e., *N*_e_, effective population size; *T*, divergence time in years; 2*N*_e_m, population migration rate). For the estimate of *T*, *t* must be divided by the geometric mean of mutation rate per year per locus. Unfortunately, it is difficult to estimate mutation rate of *Shorea* species due to the absence of precisely dated fossil records. Thus, the minimum and maximum mutation rates per site per year for synonymous nucleotide substitutions in nuclear genes studied in other tree species: *u*_syn_ = 0.7 × 10^−9^ in *Pinus* (Willyard et al. 2007) and *u*_syn_ = 2.61 × 10^−9^ in palms (Gaut et al. 1996) were used as the mutation rate for silent sites (intron and synonymous sites) in *S. parvifolia*. Mutation rate per site per year for nonsynonymous site was computed by multiplying synonymous mutation rate by the observed *K*_a_/*K*_s_ ratio. Eventually, the minimum and the maximum of the calculated geometric means of the mutation rates per locus per year for *S. parvifolia* were 2.58 × 10^−7^ and 9.63 × 10^−7^_,_ when five loci were included, and 2.87 × 10^−7^ and 1.07 × 10^−6^_,_ when *GBSSI* locus was excluded from analysis. To obtain the estimates of *N*_e_, θ should be divided by 4*V* where *V* is mutation rate per locus per generation. Assuming minimum generation time for *Shorea* as 60 years (Ashton 1969), the minimum and the maximum mutation rates per locus per generation (*V*) for *S. parvifolia* were computed as 1.55 × 10^−5^ and 5.78 × 10^−5^ for five loci, and 1.72 × 10^−5^ and 6.42 × 10^−5^ for four loci excluding the *GBSSI* gene. Population migration rate 2*N*_e_*m* per generation was computed by multiplying θ by *m*/2.

To test several different demographic models, log-likelihood ratio (LLR) tests between the full model and nested models were performed using the results of simulations performed using IMa program. These tests are also implemented in the IMa program (Hey and Nielsen 2007). The full model includes all six parameters estimated by the aforementioned simulations, while some parameters are fixed (e.g., *m*_sm_ = 0) in the nested models. For one of the nested models, *m*_sm_ = *m*_b_ = 0, namely no migration after divergence (isolation model), the LLR is expected to follow a mixed χ^2^ distribution. However, this is not a good approximation (IM discussion group: http://groups.google.com/group/isolation-with-migration). Therefore, deviations of the LLR of the full and nested models (*m*_sm_ = *m*_b_) from a χ^2^ distribution (df = 1) were tested first. Subsequently, the deviation of the LLR of the nested models (*m*_sm_ = *m*_b_) and (*m*_sm_ = *m*_b_ = 0) was tested from a mixed χ^2^ distribution: one half of the values is zero and the other half follows a χ^2^ distribution (df = 1). If neither test was rejected the isolation model was accepted as a null model.

## Results

### Sequences obtained in this study

Sequences of the partial regions of the five nuclear genes, *GapC*, *GBSSI*, *PgiC*, *SBE2*, and *SODH*, were obtained for 78 individuals of *S. parvifolia* from seven populations, and a single individual of each *S. acuminata*, *S. leprosula*, *S. curtisii*, and *S. maxwelliana*. As sequences of both haplotypes for each individual were determined, in total, we obtained 156 sequences of *S. parvifolia* and two sequences of each individual of other species for each gene region. The alignment length was 1111, 1229, 1248, 1075, and 1277 bp, respectively (5940 bp in total).

For the *SODH* gene region, three haplotypes were observed in the individual of *S. maxwelliana*, suggesting the existence of more than one copy of the *SODH* gene in this species. To determine whether the *SODH* gene is duplicated in *S. maxwelliana,* we constructed NJ tree of this gene using all sequences obtained in this study. The three haplotypes from *S. maxwelliana* formed a separate group on the NJ tree, which was distinct from haplotypes of the other *Shorea* species (data not shown), indicating this gene duplication was specific to this species. *Shorea maxwelliana* was used as outgroup in the MK and HKA tests, which require that haplotypes from a single copy of a gene are used. Because it is difficult to identify which haplotype combination constitutes a genotype of a particular copy of the *SODH* gene, we considered all six possible allele combinations as genotypes of a single copy of the *SODH* gene. Namely, three heterozygous combinations, and three homozygous combinations, and tested each genotype in the neutrality tests. Results for the acceptance or rejection of null hypothesis were the same for each genotype. Therefore, one of these genotypes was used in further analyses. In *S. parvifolia*, only two haplotypes were observed at any locus indicating that only a single gene copy was amplified for all loci.

Of the 78 *S. parvifolia* individuals investigated, one putative F1 hybrid between *S. parvifolia* and *S. acuminata* was found in population Nanjak Makmur from Sumatra. This individual was a heterozygote harboring one *S. parvifolia* haplotype and one *S. acuminata* haplotype at all five investigated loci. Haplotype sharing with different species was not observed at any locus in other individuals. Because inclusion of a putative hybrid will bias estimation of the summary statistics, the aforementioned putative hybrid was excluded and consequently 154 sequences of *S. parvifolia* were used for all further analyses.

### Nucleotide polymorphism and recombination rate

Average haplotype diversity (*Hd*), nucleotide diversity (π), and population recombination rate per site (ρ) over five loci are summarized in Table 1. These statistics for individual loci are summarized in Table S3 together with other parameters. Nucleotide diversity for silent sites (π_sil_) varied about twofold between the five investigated loci, from 0.0053 (*GapC*) to 0.0113 (*GBSSI*), with an average of 0.0075 for a total sample of *S. parvifolia*. For the nonsynonymous sites (π_rep_), it ranged from 0.0001 (*SBE2*) to 0.0014 (*GBSSI*), with an average of 0.0006, which is more than 10-fold smaller than silent polymorphism. The levels of polymorphism also differed among populations. Especially, populations Seremban and Mersing from peninsular Malaysia had slightly lower levels of nucleotide diversity (π) and haplotype diversity (*Hd*) compared to the other populations included in our present study.

**Table 1:** Average of the estimated summary statistics over five loci

Population	*n*	*Hd*	Total 5628 bp π__total_	Silent 4272.69 bp π__silent_	Synonymous 407.69 bp π__syn_	Replacement 1344.31 bp π__rep_	ρ
Seremban	24	0.833	0.0045	0.0060	0.0053	0.0004	0.0023
Mersing	24	0.800	0.0033	0.0044	0.0043	0.0004	0.0014
Nanjak Makmur	22	0.910	0.0048	0.0061	0.0067	0.0006	0.0009
Asialog	24	0.867	0.0058	0.0074	0.0060	0.0008	0.0033
Sari Bumi Kusuma	24	0.953	0.0064	0.0080	0.0088	0.0011	0.0026
ITCI Karya Utama	12	0.903	0.0051	0.0065	0.0073	0.0003	0.0026
Sumalindo	24	0.926	0.0061	0.0076	0.0060	0.0006	0.0044
Total	154	0.949	0.0058	0.0075	0.0068	0.0006	0.0084

*n*, number of sequences; *Hd*, haplotype diversity (Nei 1987); π, nucleotide diversity (Nei 1987), total number of sites analyzed is indicated in the upper row for each type of sites; ρ, population recombination parameter estimated using the composite-likelihood method (Hudson 2001).

Similar to nucleotide diversity, population recombination rate (ρ) per site differed among the five loci as well as among populations. For the total sample of *S. parvifolia*, ρ ranged from 0.0009 (*PgiC*) to 0.0262 (*SODH*). In comparisons among populations, ρ was low in populations Seremban and Mersing from peninsular Malaysia and population Nanjak Makmur from Sumatra. In population Mersing, ρ was 0 except for the *SODH* gene region. The lower levels of nucleotide diversity and recombination rate in populations from peninsular Malaysia and Sumatra suggested smaller effective population size of those populations.

### Neutrality tests under the standard neutral model

Results of Tajima's neutrality tests for *D* statistics are summarized in Table 2. For the total sample of *S. parvifolia*, significant negative Tajima’*D* values were observed in all five loci. At population level, the values of Tajima's *D* were negative in almost all loci and populations and some were significantly negative. Such genome-wide patterns indicated the influences of demographic factors rather than locus specific factors such as natural selection. The MK test detected significantly higher nonsynonymous to synonymous ratio for polymorphism (29/23) than for divergence (1/12) in the *GBSSI* gene region (Table 3), suggesting excess of non-synonymous polymorphism at this locus. In the HKA test, there was no significant difference in the ratio of polymorphism to divergence among individual loci (data not shown).

**Table 2:** Summary of Tajima's *D* test statistics

Population	*GapC*	*GBSSI*	*PgiC*	*SBE2*	*SODH*	Average
Seremban	–0.669	–0.822	–0.394	–1.851*	0.102	–0.727
Mersing	0.666	–0.501	–1.213	–0.632	–0.771	–0.490
Nanjak Makmur	–0.394	–0.959	–2.232**	–1.145	0.585	–0.829**
Asialog	–0.371	–1.262	–0.382	–0.136	–0.812	–0.593
Sari Bumi Kusuma	–1.153	–1.351	–1.734	–1.629	–1.585	–1.490***
ITCI Karya Utama	0.478	–0.438	–1.869*	–0.252	–0.143	–0.445
Sumalindo	–1.668	–1.683	–1.762	–0.930	–1.293	–1.467***
Total	–1.762*	–2.111*	–2.247**	–1.784*	–2.007*	–1.982***

Sumatra-Malay group	–0.940	–1.756*	–1.772*	–1.410*	–1.033	–1.382***
Borneo group	–1.593*	–1.863**	–2.036**	–1.587*	–1.729*	–1.761***

* *P* < 0.05,

** *P* < 0.01

*** *P* < 0.001.

**Table 3:** Results of the MK test

		Number of variable sites			
				
Locus		Fixed	Polymorphic	Fisher's exact test (*P*-value)	G test (*P*-value)
*GapC*	Synonymous	3	10		
				0.12874	0.06825
	Nonsynonymous	4	2		
*GBSSI*	Synonymous	12	23		
				0.00181	0.00078
	Nonsynonymous	1	29		
*PgiC*	Synonymous	2	13		
				1.00000	0.79974
	Nonsynonymous	1	9		
*SBE2*	Synonymous	1	4		
				0.52381	0.34063
	Nonsynonymous	2	2		
*SODH*	Synonymous	1	7		
				0.21678	0.06971
	Nonsynonymous	3	2		

### Population subdivision

**Figure 3:**
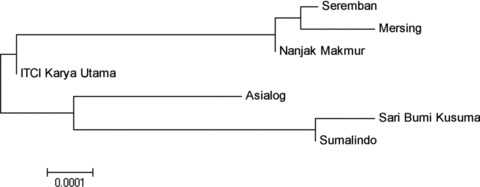
NJ tree showing relationships of the analyzed populations. Distances between the populations were estimated by the net number of nucleotide differences (*Da*; Nei 1987) using the concatenated sequences over the five loci.

**Figure 4:**
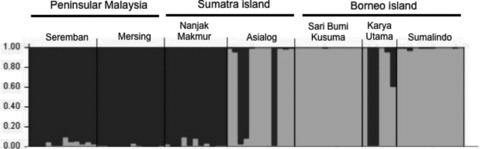
Clustering results for *S. parvifolia* individuals obtained from the STRUCTURE analysis under the admixture model with two clusters. Each individual is represented by a single column, which is partitioned into one or two colored segments that represent the individual's estimated membership fractions in the clusters.

Relatively low levels of population differentiation within peninsular Malaysia populations (*F*_ST_ < 0.107) and within Borneo populations (*F*_ST_ < 0.169) were observed (Table S5). On the other hand, high levels of population differentiation (*F*_ST_ > 0.250) were observed at some loci between populations Nanjak Makmur and Asialog from Sumatra, between peninsular Malaysia and Borneo populations, and between Sumatra and Borneo populations. The NJ tree based on the *Da* values revealed two major groups of populations (Fig. 3). First group included populations Seremban, Mersing (from Malay peninsula), Nanjak Makmur (from Sumatra), and ITCI Karya Utama (from Borneo). The other group, contained populations Asialog (from Sumatra), Sari Bumi Kusuma, and Sumalindo (from Borneo). To further evaluate population structure, model-based clustering (STRUCTURE analysis) was performed using multilocus genotype data. The Δ*K* values showed a clear peak at *K* = 2, indicating that the investigated *S. parvifolia* individuals could be split into two genetically distinct groups at the uppermost hierarchical level. When *K* = 2, all individuals from populations Seremban and Mersing in peninsula Malaysia and Nanjak Makmur in Sumatra were assigned to one cluster, and all individuals from populations Sari Bumi Kusuma and Sumalindo in Borneo were assigned to another cluster (Fig. 4). On the other hand, individuals from populations Asialog in Sumatra and ITCI Karya Utama in Borneo were assigned to either of the two clusters, suggesting these populations are admixtures of both clusters. This analysis revealed that the investigated *S. parvifolia* individuals could be divided into Sumatra-peninsular Malaysia and Borneo groups with one admixed population in each group. Hereafter, we designate the group consisting of populations from peninsular Malaysia and Sumatra as Sumatra-Malay group and the group consisting of populations from Borneo as Borneo group.

### Population parameters estimated under IM model

To infer the history of splitting event of the Sumatra-Malay and Borneo groups, estimates of six demographic parameters were obtained and converted using the minimum and the maximum mutation rates for the two datasets: all five loci (Table 4) and four loci excluding the *GBSSI* gene (Table S6). Estimates for θ per locus were smallest in the ancestral population of the two groups, followed by the Sumatra-Malay and Borneo groups. Marginal distributions of these three parameters hardly overlapped (Fig. S1A), which strongly supported the relationship of θ: θ_A_ < θ_sm_ < θ_b_. This result can be directly interpreted as the relationship of their effective population sizes *N_e_A_* < *N*_e sm_ < *N*_e b_. Converted estimates of the effective population size of the ancestral population were 18,833 and 5051 when the minimum and maximum mutation rates were used, respectively. The effective population sizes of the Sumatra-Malay and Borneo groups were about three and 10 times larger than the ancestral group, respectively. The estimate of migration rate was very small in both directions (Fig. S1B). The converted migration rate from the Borneo group to the Sumatra-Malay group (2*Nm*_sm_ = 1.65) was larger than the migration rate from the Sumatra-Malay group to the Borneo group (2*Nm*_b_ = 0.25). The marginal distribution of *t* had clear peak (Fig. S1C) and the estimates of divergence time (*t*) of the two groups were very similar between the two datasets: 0.685 and 0.635 for five loci and four loci, respectively. For the dataset with five loci, the estimated divergence time of the Sumatra-Malay and Borneo groups was 0.71 MYA and 2.65 MYA, when the maximum and minimum mutation rates were used, respectively. Estimates of the six parameters are only slightly different between the datasets with five and four loci excluding the *GBSSI* gene region (Table S6), suggesting our estimates are robust even when the *GBSSI* gene region was included.

**Table 4:** Demographic parameters estimated by the IMa program

Parameters	Estimates		(90% HPD)
θ_sm_	4.367		(2.9313 – 6.4009)
θ_b_	11.2873		(8.1736 – 15.1795)
θ_A_	1.1677		(0.5449 – 2.7245)
*T*	0.685		(0.495 – 0.945)
*m*_sm_	0.755		(0.265 – 1.485)
*m*_b_	0.045		(0.005 – 0.405)
Converted parameters	Maximum mutation rate (*u_1_*)		Minimum mutation rate (*u_2_*)

*N_e_sm_*	18,890		70,433
*N_e_b_*	48,824		182,046
*N_e_A_*	5,051		18,833
*T*	711,131		2,651,502
2*Nm_sm_*		1.65	
2*Nm_b_*		0.25	

Maximum and minimum mutation rates per site per year for synonymous site in tree species: *u_1_* = 2.61 × 10^−9^ in palms (Gaut *et al*. 1996) and *u_2_* = 0.7 × 10^−9^ in *Pinus* (Willyard *et al*. 2007) were used for conversions.

*N*_e_, effective population size of Sumatra-Malay (*sm*) and Borneo (*b*) groups and the ancestral population (A); *T*, divergence time in years; 2*Nm*, population migration rate per generation from Borneo to Sumatra-Malay groups (sm) and Sumatra-Malay to Borneo groups (b).

Results of the likelihood ratio test for a series of nested models are shown in Table S7. For the dataset with five loci, only one nested model was not rejected: the model in which *m*_b_ = 0. Isolation model (*m*_sm_ = *m*_b_ = 0) was rejected for the dataset with five loci (*P* = 10^−17^) but not for the dataset with four loci (*P* = 0.13730). The models in which a pair of or all of θ_sm_, θ_b_, and θ_A_ are the same were rejected in both datasets (*P* < 0.01), which also supported the relationship of θ_A_ < θ_sm_ < θ_b_. In addition, a clear peak in marginal distribution of the divergence time (*t*) of these groups (Fig. S1C), indicated that the migration model where migration rate is constant and *t* is essentially infinity could also be reasonably rejected.

### Neutrality tests using the inferred demographic model

To test whether the observed deviation of Tajima's *D* statistics from neutrality could be explained by demographic factors, the expected distribution of Tajima's *D* was obtained using inferred population structure and demographic model (Table S8). Namely, with two population groups (Sumatra-Malay and Borneo) and with six parameters estimated by the IMa program. The Tajima’*D* values, which were significantly negative using the standard neutral model for a total sample of *S. parvifolia* or individual population groups became all insignificant using the demographic model. This result suggested that the observed deviations from neutrality detected by the Tajima's test using the standard neutral model could be explained by demographic factors such as migrations and expansions rather than natural selection.

## Discussion

### Nucleotide polymorphism and population structure of *S. parvifolia*

In this study, the levels and patterns of nucleotide poly-morphism in five nuclear loci of *S. parvifolia* from peninsular Malaysia, Sumatra, and eastern Borneo were investigated. To our knowledge, population genetic analyses based on the sequences of multiple nuclear loci in tropical rainforest tree species at a regional scale have not been undertaken in the past. The level of nucleotide diversity of *S. parvifolia* at silent sites over five loci (π_sil_ = 0.0075) is in the lower range of those reported for herbaceous species and quite similar to values reported for other woody species (Fig. S2). It is remarkable that woody species have lower and less variable levels of π_sil_ compared to herbaceous plants, despite that a broad taxonomical range of species, with varying geographic ranges and breeding systems, were included in the comparison (Fig. S2). This suggests that there may be factors that restrict the level of genetic variation of woody species, obscuring the effects of geographic range and breeding system. This remains to be clarified by future studies.

Our previous study suggested that hybridization among closely related *Shorea* species in peninsular Malaysia could be a reason for the presence of diverged haplotypes at some loci (Ishiyama et al. 2003, 2008). In this study, only one putative hybrid between *S. parvifolia* and *S. acuminata* was found. Therefore, the effects of hybridization on the level of polymorphism in *S. parvifolia* were probably negligible. However, from the point of view of the evolution of *S. parvifolia*, hybridization could not be neglected as a source of new alleles even if it is rare and local, especially for populations with small effective population size.

Considerable levels of population differentiation (*F*_ST_ > 0.25) were observed between peninsular Malaysia or Sumatra and Borneo populations. STRUCTURE analysis revealed that the investigated *S. parvifolia* individuals could be divided into two genetically distinct groups: Sumatra-Malay and Borneo with one admixed population in Sumatra (Asialog) and one admixed population in Borneo (ITCI Karya Utama). Clear separation of the two groups except for one individual from ITCI Karya Utama (Fig. 4) suggests limited genomic exchanges between them and that the admixture occurred quite recently or reproductive isolation has developed between the two groups. Analysis of *S. parvifolia* populations based on AFLP data (Cao et al. 2006) revealed that they were also separated into two groups. Furthermore, they found another admixed population (Pasir Mayang) located between Nanjak Makmur and Asialog (Fig. S3). In addition, all individuals from the population TNBT located in the center of Sumatra clustered with individuals from Borneo (Fig. S3), suggesting that a large area was involved in the admixture event and that *S. parvifolia* has complicated population structure.

### Deviation from neutrality and evolution of *S. parvifolia*

In the MK test, excess of nonsynonymous polymorphism was detected in the *GBSSI* gene region (Table 3). In populations from Sumatra and Borneo, significant negative Tajima's *D* values were observed only at nonsynonymous sites in this gene region (Table S4), indicating that nonsynonymous variants were present at low frequency. It thus appears that nonsynonymous mutations in the *GBSSI* gene region are slightly deleterious and probably in the process of being eliminated by purifying selection, as predicted to occur in populations with large effective population size (*N_e_s* > 1; Ohta 1976). On the other hand, in populations Seremban and Mersing from peninsular Malaysia, the values of Tajima's *D* were close to 0 or positive at nonsynonymous sites of the *GBSSI* gene region (Table S4). This observation could be attributed to a smaller effective population size of these populations, which allows slightly deleterious mutations to be maintained in populations or fixed by random genetic drift (Ohta 1976).

How big fraction of *S. parvifolia* nuclear genome has pattern of variation similar to that of the *GBSSI* gene, namely slightly deleterious mutations have elevated frequency in populations from peninsular Malaysia, is an interesting question in terms of genetic conservation. This kind of information can be used for evaluation of the levels of genetic degradation and for the assessment of the extinction risks. Obviously, this question is also relevant for the understanding speciation of the genus *Shorea*, which contains very large number of species (approximately 190) in Southern and Southeastern Asia.

### Demographic history of *S. parvifolia* and climatic changes

**Figure 5:**
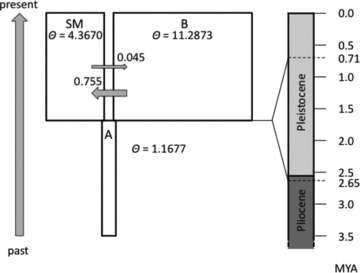
Schematic illustration of the six demographic parameters estimated under the IM model. The width of the white rectangles representing Sumatra-Malay group (SM), Borneo group (B), and the ancestral population (A) are scaled by the estimated θ for each group. Estimated migration rates in each direction are indicated by arrows. The range of converted divergence time of Sumatra-Malay and Borneo groups is shown with the geological time scale on the left side.

Divergence history of the Sumatra-Malay and Borneo groups was inferred by IMa analysis. Estimates of the six demographic parameters are only slightly different between the datasets with five and four loci excluding the *GBSSI* gene region (Table S6). We therefore focus on results obtained with the dataset including five loci. Schematic representation of the estimated six demographic parameters is illustrated in Figure 5. Assuming that the mutation rate of *S. parvifolia* is in the range known for the other tree species, the divergence time of the Sumatra-Malay and Borneo groups is in the range from about 2.7 to 0.7 MYA, which corresponds to the late Pliocene and the middle Pleistocene respectively. In addition, about threefold and 10-fold larger effective population size in the Sumatra-Malay and Borneo groups, respectively, than that of the ancestral population suggested population expansion of the two groups after their divergence. In fact, multi-locus trend toward negative Tajima's *D* in all populations suggested a regional scale of population expansion (Table 2).

Divergence and expansions of the two groups were probably caused by climatic changes from the late Pliocene onwards. From the early Miocene to late Pliocene (23 ∼ 2.6 MYA), conditions of Southeastern Asia were warm and humid with widespread rainforest (Morley 2000), and sea levels were higher than today (see Woodruff 2010 and references therein), which kept peninsular Malaysia, Sumatra, Java, Borneo, and other small islands separated by the ocean. Mean sea levels started to decline (–16 ± 10 m) gradually from 2.6 MYA in the late Pliocene with profound fluctuations coinciding with the glacial cycles (Woodruff 2010). Peninsular Malaysia, Sumatra, Java, and Borneo were connected by the exposed Sunda Shelf, when the sea level dropped and savanna vegetation appeared (Morley 2000). Taking into account such climatic history, and our demographic inferences, it appears that until late Pliocene the ancestral population of the investigated populations contracted in certain areas. The exposed Sunda Shelf in the Pleistocene allowed for their spread. However, simultaneous appearance of savanna vegetation in the central part of the Sunda Shelf split the ancestral population into Sumatra and Borneo regions.

Population expansion of the Sumatra-Malay and Borneo groups after their divergence indicated an abundance of favorable habitats during Pleistocene such as moist and warm forests in these areas. In fact, recent study revealed that southwest Sumatra and substantial region of Sunda Shelf and Borneo were covered by rainforests during much of the last million years (Cannon et al. 2009). In the same study, it was suggested that rainforest area in Sumatra was considerably smaller than that on the Sunda Shelf and Borneo, which is consistent with the difference in the effective population size between Sumatra-Malay and Borneo groups revealed in our present study. Two peninsular Malaysia populations (Seremban and Mersing) were collected from the area, which is supposed to have been covered by savanna at the LGM (approximately 0.02 MYA; Gathorne-Hardy et al. 2002). Genetically close relationship between these two populations and one population from Sumatra (Nanjak Makmur; Fig. 4; Table S5), and lower nucleotide diversity suggest that these populations have expanded into this area from refugia in Sumatra after the last glacial period.

In the LLR tests, the isolation model was rejected when all five loci were included, suggesting that there were migrations after divergence of the Sumatra-Malay and Borneo groups. *Shorea parvifolia* has been reported to be pollinated by thrips (Appanah and Chan 1981) or beetles (Sakai et al. 1999), which are not likely to disperse pollen for long distances. Seeds of Dipterocarpaceae are dispersed by gravity and there is no evidence that they can withstand sea water (Ashton 1982). These facts suggest that migration between the two population groups must have occurred when their distributions were close enough to allow for pollen and/or seed exchange. Based on the geographic distribution of the admixed populations (Fig. S3), the contact zone would have ranged from western Sumatra to southern Borneo. However, very low migration rates in both directions (2*N*_e_*m* per generation <2) revealed in our present study, imply that contacts of the two population groups were limited. Therefore, when Sunda Shelf was exposed, the expanding savanna must have been strong but imperfect barrier to gene flow between the two groups. In summary, our results suggest the existence of a temporary savanna corridor across Sunda Shelf during the glacial periods of the Pleistocene, and a predominance of tropical lowland rainforest at least in Sumatra and eastern Borneo.

### Comparisons with other rainforest species in Southeastern Asia

Phylogeographic studies have been done for some rain-forest plant species and animals. Penninsular Malaysia and Borneo populations of a montane tree *Trigonobalanus verticillata* were suggested to have diverged around 17 MYA in Pliocene (Kamiya et al. 2002), which precedes by more than 10 MYA the divergence of the two groups of lowland rainforest tree *S. parvifolia* revealed in our present study. This gives evidence of independent divergence histories in montane and lowland rainforest tree species, indicating different response of individual species to past climatic changes in this region.

In contrast, population structure and divergence history similar to *S. parvifolia* have been shown in some lowland rainforest species. Comparable genetic differences between Sumatra and Borneo populations were detected in *S. parvifolia* and *S. leprosula* (Cao et al. 2006). The deep split between Borneo and other Sunda lineages was also demonstrated for the rainforest-dependent rodents (Gorog et al. 2004), where the estimated divergence time among the island populations was in the range of 0.6 to 4.7 MYA, which is similar to the estimate for *S. parvifolia* obtained in our present study. All this suggests they could share the same divergence event. However, contrary to *S. parvifolia*, no evidence for admixed populations was found in *S. leprosula* (Cao et al. 2006) and rodents (Gorog et al. 2004). While dispersal between peninsular Malaysia and Borneo populations presumably across Sunda Shelf during Pleistocene was detected for some species of the pioneer trees from the genus *Macaranga* (Bänfer et al. 2006). Although no evidence for migration between peninsular Malaysia and eastern Borneo was found in the present study, migration would be possible between peninsular Malaysia and western Borneo populations via the exposed Sunda Shelf. Regrettably, our sampling in Borneo was limited to the central and eastern parts of the island. As revealed by a recent study, populations of two pioneer *Macaranga* species from eastern and western Borneo were highly differentiated (Guicking et al. 2011). It is therefore possible that *S. parvifolia* populations from Borneo are also genetically structured. More studies including wider sampling are required to address this issue.

## Conclusions

In this study, we detected two major genetically different groups of populations: Sumatra-Malay and Borneo. We estimated that they diverged sometime between late Pliocene and middle Pleistocene. Therefore, the appearance of savanna vegetation on the exposed Sunda Shelf in the Pleistocene was likely to have separated these two groups. Results of LLR tests strongly supported expansion of these two groups and low level of migration after their divergence, which in turn suggests the existence of a scarcely forested land bridge connecting Sumatra and Borneo and a predominance of tropical lowland rainforest (at least in Sumatra and eastern Borneo) during the glacial periods in the Pleistocene. Contrasting pattern of natural selection acting on nonsynonumous mutations between populations from peninsular Malaysia and other regions was also suggested, which is likely due to differences in the effective population size. Analysis of additional gene regions and populations will enable to test more complicated demographic models such as a model with different timings of population expansion between two groups. Application of similar methods to other tropical tree species in Southeastern Asia would reveal more detailed picture of biogeographic history of this region.

## Supporting Information

**Figure S1.** Marginal posterior probability distributions for model parameters obtained in the IMa analysis (see text for details).

**Figure S2.** Comparisons of nucleotide diversity at silent sites in *S. parvifolia* with those observed in herbaceous and woody plants.

**Figure S3.** Locations and clustering assignments of *S. parvifolia* populations investigated in this study and in Cao *et al*. 2006 on the modeled vegetation map for LGM.

**Table S1.** Geographic location and sample size for the investigated *Shorea* species.

**Table S2.** Sequences of primers for PCR, nested PCR and sequencing.

**Table S3.** Summary statistics for the levels of polymorphism and recombination.

**Table S4.** Summary of Tajima's D statistics.

**Table S5.** Pairwise Fst statistics between populations for individual loci.

**Table S6.** Estimates of demographic parameters obtained by the IMa program for the five and the four loci datasets.

**Table S7.** Tests of nested models for Sumatra-Malay group versus Borneo group for the five loci dataset.

**Table S8.** Lower and upper limits of Tajima's D statistic distributions under the inferred demographic model.
