# Supplementary material for: Population structure and demographic history of a tropical lowland rainforest tree species Shorea parvifolia (Dipterocarpaceae) from Southeastern Asia
Source: Ecol Evol. 2012 Jul;2(7):1663–75. doi: 10.1002/ece3.284 (PMC3434916; doi:10.1002/ece3.284)
Supplement: Supplementary file 1 [file ece30002-1663-SD2.doc]

**Supporting information**

Figure S1. Marginal posterior probability distributions for model parameters obtained in the IMa analysis (see text for details)

a) 4*N_e_u* of Sumatra-Malay group (*θ*_sm_), Borneo group (*θ*_b_) and their ancestral population (*θ*_A_)

Figure S1. continued

b) Migration rates from Borneo group to Sumatra-Malay group (*m*_sm_) and from Sumatra-Malay group to Borneo group (*m*_b_)

c) Divergence time (*t*) of Sumatra-Malay group and Borneo group

Figure S2. Comparisons of nucleotide diversity at silent sites in *S. parvifolia* with those observed in herbaceous and woody plants


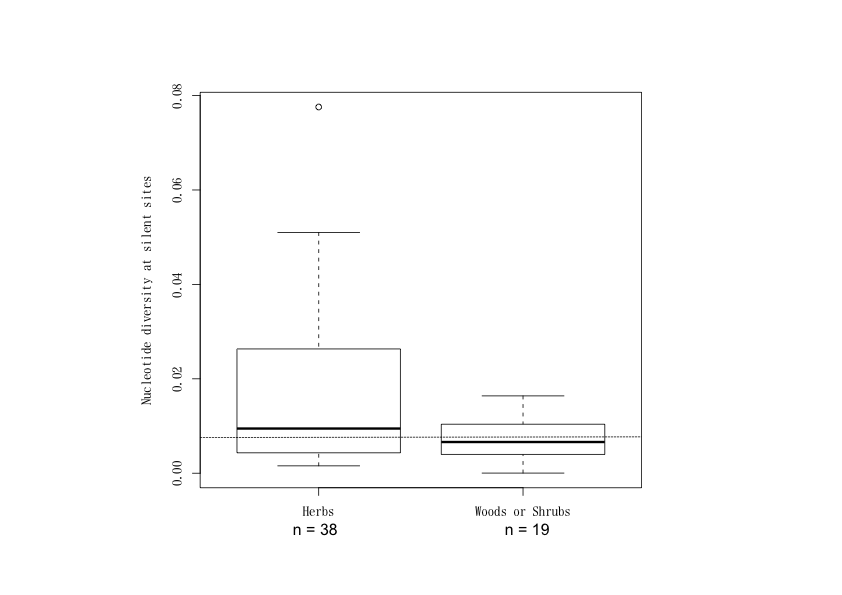


Data for herbaceous plants were obtained from Table S3 in Lynch (Mol. Biol. Evol. 2006, 23) (38 independent studies for 30 species). For woody and shrub species, additional data (see below) were integrated with the data from Table S3 in Lynch (2006) (In total: 19 independent studies for 17 species). Boxes denote the interquartile range (IQR) between the first and third quartiles (25th and 75th quartiles, respectively) and the thick line inside denotes the median. Whiskers denote the lowest and highest values within 1.5 times IQR from the first and third quartiles, respectively. Circle denotes outlier beyond the whiskers. Horizontal dashed line represents nucleotide diversity at silent sites in *S. parvifolia* (*π*_sil_ = 0.0075).

Figure S2. continued

Additional data of nucleotide diversity at silent sites for woody species

|  | n | Direct estimate | Corrected estimate | Reference |
| --- | --- | --- | --- | --- |
| *Rhizophora apiculata* | 55 | 0.00059 | 0.00060 | Inomata *et al*. (2009, Genes Genet. Syst. 84: 277-286) |
| *Rhizophora mucronata* | 62 | 0.00003 | 0.00003 | Inomata *et al*. (2009, Genes Genet. Syst. 84: 277-286) |
| *Zanthoxylum ailanthoides* | 88 | 0.0056 | 0.00566 | Kamiya *et al*. (2008, Mol. Ecol. 17: 2329-2338) |
| *Zanthoxylum schinifolium* | 12 | 0.0032 | 0.00349 | Kamiya *et al*. (2008, Mol. Ecol. 17: 2329-2338) |
| *Pinus densata* | 66 | 0.0122 | 0.01239 | Ma *et al*. (2006, Mol. Biol. Evol. 23: 807–816) |
| *Pinus tabuliformis* | 43 | 0.0119 | 0.01218 | Ma *et al*. (2006, Mol. Biol. Evol. 23: 807–816) |
| *Pinus yunnanensis* | 29 | 0.0095 | 0.00984 | Ma *et al*. (2006, Mol. Biol. Evol. 23: 807–816) |
| *Pseudotsuga menziesii* | 30 | 0.01055 | 0.01091 | Krutovsky *et al*. (2005, Genetics. 171: 2029-2041) |
| *Cryptomeria japonica* | 18 | 0.00382 | 0.00404 | Kado *et al*. (2008, Tree Genetics & Genomes. 4: 133-141) |
| *Chamaecyparis obtusa* | 18 | 0.00689 | 0.00730 | Kado *et al*. (2008, Tree Genetics & Genomes. 4: 133-141) |
| *Chamaecyparis pisifera* | 5 | 0.00675 | 0.00844 | Kado *et al*. (2008, Tree Genetics & Genomes. 4: 133-141) |

Direct estimates were corrected by multiplying by *n*(*n*-1), where *n* is the number of sequences

Figure S3. Locations and clustering assignments of *S. parvifolia* populations investigated in this study and in Cao *et al*. 2006 on the modeled vegetation map for LGM


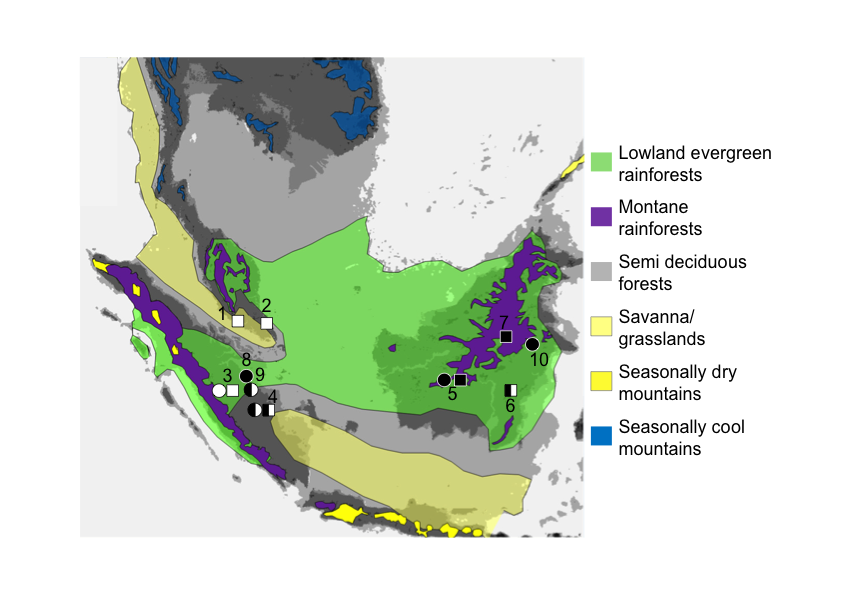


A modeled vegetation map for LGM with closed corridor scenario is adapted from Figure S1 of Cannon *et al*. 2009. The ID numbers 1 – 7 of the populations are the same as in Figure 1. Additional populations investigated by Cao *et al*. (2006): 8. TNBT, 9. Pasir Mayang, 10. Batu Ampar. Populations 3, 4 and 5 were investigated both in this study and by Cao *et al*. (2006). The admixed populations detected in this study are indicated by half solid squares. Populations belonging to the Sumatra-Malay group and Borneo group except for the admixed populations are indicated by open and solid squares respectively. Populations corresponding to the Sumatra-Malay and Borneo groups and admixed populations revealed by AFLP (Cao et al. 2006) are indicated by open, solid and half-solid circles respectively.
