# Supplementary material for: Population structure and demographic history of a tropical lowland rainforest tree species Shorea parvifolia (Dipterocarpaceae) from Southeastern Asia
Source: Ecol Evol. 2012 Jul;2(7):1663–75. doi: 10.1002/ece3.284 (PMC3434916; doi:10.1002/ece3.284)
Supplement: Supplementary file 2 [file ece30002-1663-SD1.doc]

Table S1. Geographic location and sample size for the investigated *Shorea* species

Number of

Approximate location

| Species | Population | individuals | Latitude | Longitude |
| --- | --- | --- | --- | --- |
| *S. parvifolia* | Seremban | 12 | 2° 28' 05" N | 101° 54' 16" E |
|  | Mersing | 12 | 2° 30' 60" N | 103° 36' 31" E |
|  | Nanjak Makmur | 12 | 0° 18' 59" S | 101° 50' 27" E |
|  | Asialog | 12 | 2° 01' 60" S | 103° 14' 60" E |
|  | Sari Bumi Kusuma | 12 | 0° 49' 57" S | 112° 51' 32" E |
|  | ITCI Karya Utama | 6 | 1° 31' 47" S | 116° 06' 33" E |
|  | Sumalindo | 12 | 1° 12' 13" N | 115° 11' 22" E |
|  | Total | 78 |  |  |
| *S. acuminata* | Seremban | 1 | 2° 28' 05" N | 101° 54' 16" E |
| *S. curtisii* | Mersing | 1 | 2° 30' 60" N | 103° 36' 31" E |
| *S. leprosula* | Seremban | 1 | 2° 28' 05" N | 101° 54' 16" E |
| *S. maxwelliana* | FRIM arboretum | 1 | 3° 08' 18" N | 101° 34' 45" E |

ITCI Karya Utama: International Timber Corporation Indonesia Karya Utama

FRIM: Forest Research Institute Malaysia

Table S2. Sequences of primers for PCR, nested PCR and sequencing

PCR primers

| Locus | Primer name | Direction | Sequence | Reference |
| --- | --- | --- | --- | --- |
| *GapC* | gapc-0F  gapc-int9R | F  R | TATGACCACCGTTCACTCCA  TGTGTGACGAAATGAAGACCA | in this study  in this study |
| *GBSSI* | gbssi-dipF  gbssi-1255R | F R | GTGGTCTGCCACCTGCAAT GCAGAGTAGGATTAGGGAGTGA | K. Kamiya (personal communication)  in this study |
| *PgiC* | pgic-F3n pgic-R | F R | GATCAGCATTTCTATTCAGCACC TCCTTGTGCAACTCTTCTGG | in this study in this study |
| *SBE2* | sbe2-exo7F  sbe2-3585R | F R | GGAATGAGTAGCACGGTATGC ACACCATCAAATCTAAATCCATCA | in this study in this study |
| *SODH* | sodh-exo2F  sodh-int2.5R | F R | TGTTGGTATCTGCGGAAGTG TGCATGTGTGTTCGGTTTAT | in this study in this study |
| Nested PCR primers | | | | |
| Locus | Primer name | Direction | Sequence | Reference |
| *GapC* | gapc-0F  gapc-1119R | F  R | TATGACCACCGTTCACTCCA  TCAAAATCAAACTAAGGTGCTG | in this study  in this study |
| *GBSSI* | gbssi-14F  gbssi-1255R | F R | TGCAATGGCGGTGAGAAA GCAGAGTAGGATTAGGGAGTGA | in this study in this study |
| *PgiC* | pgic-F  pgic-R | F R | CAGCATTTCTATTCAGCACCTTT TCCTTGTGCAACTCTTCTGG | in this study in this study |
| *SBE2* | sbe2-exo7F  sbe2-int10R | F R | GGAATGAGTAGCACGGTATGC TGCATACTTCCCAACTTCCA | in this study in this study |
| *SODH* | sodh-exo2F  sodh-int3-1R | F R | TGTTGGTATCTGCGGAAGTG CCAAGGTGATGATGAAGACAA | in this study in this study |

Table S2. Continued

Sequencing primers

| Locus | Primer name | Direction | Sequence | Reference |
| --- | --- | --- | --- | --- |
| *GapC* | gapc-0F  gapc-int8-1F gapc-int8-2F gapc-1119R gapc-int8-1R gapc-808R | F  F F R R R | TATGACCACCGTTCACTCCA  TGCAGGGCCAACAAAATTA TGCCTGCATTATTAGCAACTG TCAAAATCAAACTAAGGTGCTG GGCCCTGCAAACTAGCATAA CCCTGAAAACAACCCAGATACAA | in this study  in this study in this study in this study in this study in this study |
| *GBSSI* | gbssi-14F gbssi-dip1F gbssi-dip2F gbssi-1255R gbssi-dip3R gbssi-dip2R | F F F R R R | TGCAATGGCGGTGAGAAA TCAGTTTGATGTTGGAATTGG GGCATACTGCCCTTCTTCC GCAGAGTAGGATTAGGGAGTGA GATTTTGGATCCCGTTTTCC CAGGGAAGAAGGGCAGTATG | in this study  K. Kamiya (personal communication) K. Kamiya (personal communication) in this study  K. Kamiya (personal communication) K. Kamiya (personal communication) |
| *PgiC* | pgic-F pgic-F4 pgic-F7 pgic-R pgic-R8n pgic-R6 | F F F R R R | CAGCATTTCTATTCAGCACCTTT ATTTGTGTTGGCTAACCCTC GACGTGTAATTCCATGTGATTT TCCTTGTGCAACTCTTCTGG AATGAGGAGCTCAAGAATCA CTTTAAGGTGGATAGGTTGCTG | in this study in this study  Ishiyama et al. (2008)  in this study in this study  Ishiyama et al. (2008) |
| *SBE2* | sbe2-int7-0F sbe2-int7-2F sbe2-int8F sbe2-int9R sbe2-exo8R sbe2-int7R | F F F R R R | TGCTTGTGCTGAACATCATATTG TATGCCCCCATTCAAATAAC TGGAATGGGACAGGTTTTCA ATTTTCTCTGGCCAATCTGA GCCTTTTAATTCGAGGAAGCA TTATGCCTTCGAATTTGTCC | in this study in this study in this study in this study in this study in this study |
| *SODH* | sodh-exo2F sodh-exo3-1F sodh-790F sodh-int3-1R sodh-exo3-1R sodh-850R | F F F R R R | TGTTGGTATCTGCGGAAGTG CTATGGTGATTGGGCATGAG GCAGGTGGTATAAAATCTCCAG CCAAGGTGATGATGAAGACAA CTTCACCTCACTGCCAACCT TTTTGCAGTTGCGTGACTTT | in this study in this study in this study in this study in this study in this study |

Table S3. Summary statistics for the levels of polymorphism and recombination

Number of

Total

Silent Synonymous Replacement

Locus Population *n*a *S*c haplotypes *Hd* *θw* *π*_total *π*_silent *π*_syn *π*_rep *ρ*

|  | | | | | | 1059.00 | 813.16 | 79.16 | 241.84 |  |
| --- | --- | --- | --- | --- | --- | --- | --- | --- | --- | --- |
| Seremban | 24 | 14 | 10 | 0.848 | 0.0035 | 0.0028 | 0.0036 | 0.0049 | 0.0000 | 0.0014 |
| Mersing | 24 | 8 | 6 | 0.728 | 0.0020 | 0.0024 | 0.0031 | 0.0059 | 0.0000 | 0.0000 |
| Nanjak Makmur | 22 | 16 | 11 | 0.918 | 0.0041 | 0.0036 | 0.0046 | 0.0057 | 0.0000 | 0.0003 |
| Asialog | 24 | 22 | 13 | 0.92 | 0.0056 | 0.0050 | 0.0063 | 0.0085 | 0.0003 | 0.0013 |
| Sari Bumi Kusuma | 24 | 26 | 17 | 0.938 | 0.0065 | 0.0045 | 0.0056 | 0.0080 | 0.0007 | 0.0036 |
| ITCI Karya Utama | 12 | 11 | 8 | 0.924 | 0.0034 | 0.0038 | 0.0050 | 0.0021 | 0.0000 | 0.0032 |
| Sumalindo | 24 | 22 | 14 | 0.888 | 0.0055 | 0.0030 | 0.0039 | 0.0011 | 0.0000 | 0.0011 |
| Total | 154 | 56 | 51 | 0.953 | 0.0094 | 0.0042 | 0.0053 | 0.0063 | 0.0002 | 0.0049 |

*GapC*

*GBSSI* 1144.00 724.01 125.01 417.99

| Seremban | 24 | 36 | 17 | 0.971 | 0.0083 | 0.0067 | 0.0100 | 0.0061 | 0.0008 | 0.0034 |
| --- | --- | --- | --- | --- | --- | --- | --- | --- | --- | --- |
| Mersing | 24 | 36 | 11 | 0.866 | 0.0081 | 0.0070 | 0.0101 | 0.0072 | 0.0014 | 0.0000 |
| Nanjak Makmur | 24 | 47 | 15 | 0.953 | 0.0106 | 0.0075 | 0.0110 | 0.0104 | 0.0010 | 0.0018 |
| Asialog | 24 | 57 | 19 | 0.975 | 0.0135 | 0.0091 | 0.0130 | 0.0077 | 0.0020 | 0.0062 |
| Sari Bumi Kusuma | 24 | 48 | 17 | 0.967 | 0.0108 | 0.0074 | 0.0104 | 0.0087 | 0.0017 | 0.0016 |
| ITCI Karya Utama | 12 | 23 | 8 | 0.924 | 0.0064 | 0.0058 | 0.0089 | 0.0096 | 0.0000 | 0.0026 |
| Sumalindo | 24 | 60 | 19 | 0.978 | 0.0137 | 0.0082 | 0.0115 | 0.0085 | 0.0023 | 0.0037 |
| Total | 154 | 137 | 91 | 0.988 | 0.0224 | 0.0077 | 0.0113 | 0.0086 | 0.0014 | 0.0075 |

*PgiC*

|  | | | | | | 1210.00 | 901.44 | 88.44 | 307.56 |  |
| --- | --- | --- | --- | --- | --- | --- | --- | --- | --- | --- |
| Seremban | 24 | 23 | 9 | 0.855 | 0.0050 | 0.0056 | 0.0073 | 0.0068 | 0.0003 | 0.0000 |
| Mersing | 24 | 15 | 9 | 0.812 | 0.0033 | 0.0021 | 0.0027 | 0.0018 | 0.0005 | 0.0000 |
| Nanjak Makmur | 22 | 41 | 12 | 0.892 | 0.0092 | 0.0041 | 0.0048 | 0.0087 | 0.0020 | 0.0000 |
| Asialog | 24 | 33 | 11 | 0.754 | 0.0072 | 0.0046 | 0.0057 | 0.0078 | 0.0013 | 0.0000 |
| Sari Bumi Kusuma | 24 | 50 | 15 | 0.942 | 0.0111 | 0.0062 | 0.0077 | 0.0202 | 0.0015 | 0.0000 |
| ITCI Karya Utama | 12 | 23 | 8 | 0.848 | 0.0064 | 0.0037 | 0.0044 | 0.0099 | 0.0016 | 0.0024 |
| Sumalindo | 24 | 38 | 13 | 0.924 | 0.0087 | 0.0047 | 0.0062 | 0.0134 | 0.0003 | 0.0004 |
| Total | 154 | 115 | 56 | 0.934 | 0.0169 | 0.0052 | 0.0066 | 0.0111 | 0.0010 | 0.0009 |

*SBE2*

|  | | | | | | 1047.00 | 861.33 | 56.33 | 183.67 |  |
| --- | --- | --- | --- | --- | --- | --- | --- | --- | --- | --- |
| Seremban | 24 | 10 | 6 | 0.551 | 0.0025 | 0.0011 | 0.0013 | 0.0000 | 0.0005 | 0.0000 |
| Mersing | 24 | 5 | 5 | 0.714 | 0.0013 | 0.0010 | 0.0012 | 0.0000 | 0.0000 | 0.0000 |
| Nanjak Makmur | 22 | 10 | 8 | 0.844 | 0.0026 | 0.0017 | 0.0021 | 0.0016 | 0.0000 | 0.0000 |
| Asialog | 24 | 17 | 9 | 0.772 | 0.0043 | 0.0043 | 0.0051 | 0.0000 | 0.0005 | 0.0007 |
| Sari Bumi Kusuma | 24 | 33 | 15 | 0.942 | 0.0084 | 0.0050 | 0.0061 | 0.0015 | 0.0000 | 0.0007 |
| ITCI Karya Utama | 12 | 17 | 7 | 0.894 | 0.0053 | 0.0050 | 0.0061 | 0.0000 | 0.0000 | 0.0005 |
| Sumalindo | 24 | 23 | 11 | 0.862 | 0.0058 | 0.0044 | 0.0050 | 0.0030 | 0.0000 | 0.0008 |
| Total | 154 | 64 | 43 | 0.902 | 0.0109 | 0.0048 | 0.0058 | 0.0009 | 0.0001 | 0.0025 |

*SODH*

*n*: Number of sequences

|  | | | | | | 1168.00 | 972.75 | 58.75 | 193.25 |  |
| --- | --- | --- | --- | --- | --- | --- | --- | --- | --- | --- |
| Seremban | 24 | 28 | 15 | 0.96 | 0.0063 | 0.0065 | 0.0077 | 0.0087 | 0.0004 | 0.0065 |
| Mersing | 24 | 23 | 10 | 0.88 | 0.0052 | 0.0041 | 0.0049 | 0.0067 | 0.0000 | 0.0069 |
| Nanjak Makmur | 22 | 26 | 13 | 0.944 | 0.0060 | 0.0069 | 0.0083 | 0.0071 | 0.0000 | 0.0027 |
| Asialog | 24 | 33 | 14 | 0.913 | 0.0075 | 0.0059 | 0.0070 | 0.0058 | 0.0000 | 0.0081 |
| Sari Bumi Kusuma | 24 | 66 | 18 | 0.975 | 0.0150 | 0.0089 | 0.0101 | 0.0056 | 0.0017 | 0.0069 |
| ITCI Karya Utama | 12 | 26 | 8 | 0.924 | 0.0073 | 0.0070 | 0.0082 | 0.0149 | 0.0000 | 0.0043 |
| Sumalindo | 24 | 66 | 19 | 0.978 | 0.0150 | 0.0101 | 0.0117 | 0.0039 | 0.0004 | 0.0160 |
| Total | 154 | 124 | 72 | 0.97 | 0.0189 | 0.0071 | 0.0084 | 0.0073 | 0.0004 | 0.0262 |

*S*: Total number of segregating sites

*Hd*: Haplotype diversity (Nei 1987)

*θw*: Watterson's theta (Watterson 1975)

*π*: Nucleotide diversity (Nei 1987)

*ρ*: 4*Nc*, Population recombination parameter estimated using the composite-likelihood method (Hudson 2001) The number of sites excluding alignment gaps is indicated in the uppermost row for each locus

Table S4. Summary of Tajima's *D* statistics

Locus Population

Tajima's *D*

Total

Silent

Synonymous Replacement

*GapC* Seremban -0.669 -0.5301 0.7763 - Mersing 0.666 0.6659 1.2318 - Nanjak Makmur -0.394 -0.2561 1.1422 - Asialog -0.371 -0.1534 -0.4053 -1.1593

Sari Bumi Kusuma -1.153 -1.0278 -0.5414 -1.5147

ITCI Karya Utama 0.478 0.4776 -1.1405 - Sumalindo -1.668 -1.6675 -1.1593 - Total -1.762 -1.7083 * -1.1250 -1.2688

*GBSSI* Seremban -0.822 -0.7786 -0.7922 -0.8899

Mersing -0.501 -0.5475 -0.9375 0.1812

Nanjak Makmur -0.959 -0.8142 -0.3081 -1.7294 *** Asialog -1.262 -1.0782 -1.2171 -1.9821 ** Sari Bumi Kusuma -1.351 -1.2381 -1.3252 -1.6535

ITCI Karya Utama -0.438 -0.4380 -1.0213 - Sumalindo -1.683 -1.6060 -1.3494 -1.7650 * Total -2.111 * -1.9697 ** -2.0277 ** -2.4361 ***

*PgiC* Seremban -0.394 0.3543 0.2258 -1.1593

Mersing -1.213 -1.1875 -0.6811 -0.6811

Nanjak Makmur -2.232 ** -2.2721 ** -1.3306 -1.2461

Asialog -0.382 -1.1835 ** -1.4315 -1.6895

Sari Bumi Kusuma -1.734 -1.7762 * -0.5278 -0.3543

ITCI Karya Utama -1.869 * -1.8049 * -0.3785 -1.6293 *** Sumalindo -1.762 -1.7369 * -0.3415 -1.1593

Total -2.247 ** -2.2148 *** -1.4834 -1.8909 **

*SBE2* Seremban -1.851 * -1.7699 * - -1.1593

Mersing -0.632 -0.6316 - - Nanjak Makmur -1.145 -1.1446 -1.1624 - Asialog -0.136 0.0947 - -1.1593

Sari Bumi Kusuma -1.629 -1.5974 -1.1593 - ITCI Karya Utama -0.252 -0.2515 - - Sumalindo -0.930 -0.9297 -1.5147 - Total -1.784 * -1.7465 * -1.7140 *** -1.3200

*SODH* Seremban 0.102 0.2015 -0.0866 -1.1593

Mersing -0.771 -0.7711 -1.2788 - Nanjak Makmur 0.585 0.5845 0.8953 - Asialog -0.812 -0.8121 0.4803 -

Sari Bumi Kusuma -1.585 -1.5979 -1.4943 -0.8899

ITCI Karya Utama -0.143 -0.0602 -0.3785 - Sumalindo -1.293 -1.2805 -0.2484 -1.1593

Total -2.007 * -2.0009 ** -1.4462 -1.1173

**P* < 0.05, ***P* < 0.01 and ****P* < 0.001

Table S5. Pairwise Fst statistics between populations for individual loci

GapC Mersing Nanjak Makmur Asialog

Sari Bumi

Kusuma Karya Utama Sumalindo

| Seremban 0.0144 0.0109 | | 0.1445 | 0.2736 | 0.1894 0.1905 | |
| --- | --- | --- | --- | --- | --- |
| Mersing | 0.0529 | 0.1986 | 0.3106 0.2620 | | 0.2164 |
| NanjakMakmur | | 0.0551 | 0.2299 0.1649 | | 0.1399 |
| Asialog | | | 0.1808 0.1557 0.1558 | | |
| Sari Bumi Kusuma 0.0218  Karya Utama | | | | | 0.0838  0.0992 |

GBSSI Mersing Nanjak Makmur Asialog

Sari Bumi

Kusuma Karya Utama Sumalindo

| Seremban 0.0151 0.0045 0.0432 | | 0.0601 0.0903 0.0611 | | |
| --- | --- | --- | --- | --- |
| Mersing 0.0457  NanjakMakmur | 0.0802 0.1107 | | 0.1554 | 0.1060 |
|  | 0.0928 0.0932 0.0721 0.0815 | | | |
| Asialog 0.0150  Sari Bumi Kusuma | | | 0.0843  0.0746 | 0.0255  -0.0075 |

Karya Utama 0.0381

PgiC Mersing Nanjak Makmur Asialog

Sari Bumi

Kusuma Karya Utama Sumalindo

| Seremban | 0.1072 | 0.0406 | 0.2968 | 0.1067 0.1043 0.1265 | | |
| --- | --- | --- | --- | --- | --- | --- |
| Mersing 0.0006  NanjakMakmur | | | 0.3408 | 0.1018 | 0.0187  0.0244 | 0.1017  0.0811 |
|  |  |  | 0.2683 | 0.0666 |  |  |
| Asialog | | | | 0.2663 0.2948 0.2748 | | |

Sari Bumi Kusuma 0.0366 0.0173

Karya Utama 0.0431

SBE2 Mersing Nanjak Makmur Asialog

Sari Bumi

Kusuma Karya Utama Sumalindo

| Seremban | 0.0517 | 0.0027 | 0.4786 0.5730 | | 0.2087 | 0.5781 |
| --- | --- | --- | --- | --- | --- | --- |
| Mersing | | 0.0739 | 0.5160 0.6045 0.2743 0.6120 | | | |
| NanjakMakmur | | | 0.4152 0.5220 | | 0.1587 | 0.5215 |
| Asialog | | | | 0.0920 0.0833 0.0663 | | |
| Sari Bumi Kusuma | | | | | 0.1691 | -0.0084 |
| Karya Utama | | | | | | 0.1464 |

SODH2 Mersing Nanjak Makmur Asialog

Sari Bumi

Kusuma Karya Utama Sumalindo

| Seremban | 0.0535 | -0.0164 0.0337 | 0.1622 | 0.0793 0.1442 | |
| --- | --- | --- | --- | --- | --- |
| Mersing 0.0414 0.0228  NanjakMakmur 0.0320  Asialog | | | 0.2430 | 0.0631 | 0.2231 |
|  |  |  | 0.1722 | 0.0584 0.1478 | |
|  |  |  | 0.1528 | 0.0109 | 0.1426 |
| Sari Bumi Kusuma | | | | 0.0893 | -0.0008 |
| Karya Utama | | | | | 0.0806 |

Fst

< 0.05

0.05-0.15

0.15-0.25

>0.25

Table S6. Estimates of demographic parameters obtained by the IMa program for the five and the four loci data sets

Five loci Four loci

| Parameter estimates | *θsm* | 4.367 | (2.9313 - 6.4009) | 4.3466 | (2.6531 - 6.4917) |
| --- | --- | --- | --- | --- | --- |
| (90% HPD) | *θb* | 11.2873 | (8.1736 - 15.1795) | 9.9025 | (6.8789 - 14.438) |
|  | *θA* | 1.1677 | (0.5449 - 2.7245) | 1.2851 | (0.378 - 2.9481) |
|  | *t* | 0.685 | (0.495 - 0.945) | 0.635 | (0.405 - 1.005) |
|  | *msm* | 0.755 | (0.265 - 1.485) | 0.475 | (0.065 - 1.335) |
|  | *mb* | 0.045 | (0.005 - 0.405) | 0.075 | (0.005 - 0.555) |

Parameter

maximum

minimum

maximum

minimum

conversions

mutation rate1 mutation rate2 mutation rate1 mutation rate2

| *Ne_sm* | 18,890 |  | 70,433 | 16,930 |  | 63,126 |
| --- | --- | --- | --- | --- | --- | --- |
| *Ne_b* | 48,824 |  | 182,046 | 38,571 |  | 143,815 |
| *Ne_A* | 5,051 |  | 18,833 | 5,006 |  | 18,664 |
| *T* | 711,131 |  | 2,651,502 | 593,613 |  | 2,213,327 |
| *2Nmsm* |  | 1.65 |  |  | 1.03 |  |
| *2Nmb* |  | 0.25 |  |  | 0.37 |  |

The known maximum and minimum mutation rate per site per year for synonymous site in tree species:

*u1* = 2.61x10-9 in palms (Gaut et.al. 1996) and *u2* = 0.7x10-9 in *Pinus* (Willyard et al. 2007) was used respectively for conversions.

*Ne*: effective population size of Sumarta-Malay (sm) and Borneo (b) groups and the ancestral population (A).

*T*: divergence time in years.

2*Nm*: population migration rate per generation from Borneo to Sumatra-Malay groups (sm) and Sumatra-Malay to

Borneo groups (b).

Table S7. Tests of nested models for Sumatra-Malay group *vs* Borneo group for the five loci data set

| Model parameters | log (*P*) | *t* | *q1* |  | *q2* | *qa* | *m1* | *m2* | df | -2LLR | *P* |
| --- | --- | --- | --- | --- | --- | --- | --- | --- | --- | --- | --- |
| Full model | -0.6849 | 0.7646 | 4.0896 |  | 12.5003 | 0.9499 | 1.0228 | 0.000102 |  |  |  |
| *msm = mb* | -2.6607 | 0.7074 | 5.0435 |  | 10.6542 | 1.2771 | 0.3956 | [ 0.3956] | 1 | 3.9517 | 0.046824 |
| *mb = 0* | -0.6856 | 0.7674 | 4.101 |  | 12.4807 | 0.9459 | 0.9994 | [ 0.0001] | 1* | 0.0015 | 0.969106 |
| *msm = 0* | -5.0567 | 0.3708 | 6.5513 |  | 10.8799 | 2.9111 | [ 0.0001] | 0.1004 | 1* | 8.7437 | 0.003107 |
| *msm = mb = 0* | -36.4711 | 0.4695 | 8.9073 |  | 10.6461 | 1.6565 | [ 0.0001] | [ 0.0001] | 2* | 71.5724 | 2.87E-16 |
| *θ_sm = θ_b* | -5.0216 | 0.4681 | 8.7763 | [ | 8.7763] | 1.701 | 0.1729 | 0.000101 | 1 | 8.6735 | 0.003229 |
| *θ_sm = θ_b = θA* | -31.0202 | 0.6603 | 5.104 | [ | 5.1040] | [ 5.1040] | 0.9808 | 0.5635 | 2 | 60.6706 | 6.69E-14 |
| *θ_sm = θ_b, msm = mb* | -6.311 | 0.7 | 6.8972 | [ | 6.8972] | 1.3133 | 0.2076 | [ 0.2076] | 2 | 11.2521 | 0.003603 |
| *θ_sm = θ_b, msm = mb = 0* | -38.641 | 0.3588 | 7.2976 | [ | 7.2976] | 1.6326 | [ 0.0001] | [ 0.0001] | 3* | 75.9122 | 2.31E-16 |
| *θ_sm = θ_b = θA, msm = mb* | -33.1028 | 0.7596 | 6.7122 | [ | 6.7122] | [ 6.7122] | 0.3799 | [ 0.3799] | 3 | 64.8358 | 5.44E-14 |
| *θ_sm = θ_b = θA, msm = mb = 0* | -111.3502 | 0.3506 | 6.9164 | [ | 6.9164] | [ 6.9164] | [ 0.0001] | [ 0.0001] | 4* | 221.3305 | 9.7E-47 |
| *θ_sm = θA* | -4.8101 | 0.5898 | 2.679 |  | 11.6569 | [ 2.6790] | 1.3701 | 0.000101 | 1 | 8.2505 | 0.004074 |
| *θ_sm = θA, msm = mb* | -9.0306 | 0.4612 | 3.731 |  | 10.1905 | [ 3.7310] | 0.6818 | [ 0.6818] | 2 | 16.6914 | 4.4E-05 |
| *θ_sm = θA, msm = mb = 0* | -74.9645 | 0.3708 | 5.4962 |  | 10.9 | [ 5.4962] | [ 0.0001] | [ 0.0001] | 3* | 148.5593 | 5.39E-32 |
| *θ_b = θA* | -25.8657 | 0.8909 | 2.91 |  | 7.1659 | [ 7.1659] | 1.0511 | 0.0544 | 1 | 50.3616 | 1.28E-12 |
| *θ_b = θA, msm = mb* | -32.5695 | 0.7602 | 6.9423 |  | 6.445 | [ 6.4450] | 0.3795 | [ 0.3795] | 2 | 63.7692 | 1.42E-14 |
| *θ_b = θA, msm = mb = 0* | -111.1569 | 0.3565 | 6.7224 |  | 7.0898 | [ 7.0898] | [ 0.0001] | [ 0.0001] | 3* | 220.9441 | 1.25E-47 |

Model parameters: only fixed parameters, indicated in square brackets in the right panel, are listed. log (*P*): posterior density of the model parameters given the data.

LLR: log-likelihood ratio of the full model and the nested model.

*P*: probability of achieving the test statistic (-2LLR) by chance under the null model.

*expected distribution of -2LLR is mixted. For df = 1*, *P* was obtained by halving *P*(χ2 ) (See Hey and Nielsen, 2007, Figure 2 and Table 1). For df = 2*, 3* and

[1]

4*, *P*(χ2 ), *P*(χ2 ) and *P*(χ2 ) were used respectively in this table. For the nested model (msm = mb = 0), see Materials and Methods in the text.

[2]

[3]

[4]

Table S8. Lower and upper limits of Tajima'*D* statistic distributions under the inferred demographic model

2.5% Lower limit 2.5% Upper limit

| Total | -2.34 | -0.95 |
| --- | --- | --- |
| Sumatra-Malay group | -2.01 | 0.16 |
| Borneo group | -2.25 | -0.46 |

Demographic parameters inferred for the five loci data set were used
